# Development and Validation of a Population Assay for the Seroprevalence of Lumpy Skin Disease

**DOI:** 10.3390/microorganisms14020373

**Published:** 2026-02-05

**Authors:** Manjunatha Reddy Gundallahalli Bayyappa, Sudeep Nagaraj, Shraddha Bijalwan, Chethan Kumar Harlipura Basavarajappa, Sathish Bhadravati Shivachandra, Baldev Raj Gulati

**Affiliations:** ICAR-National Institute of Veterinary Epidemiology and Disease Informatics (NIVEDI), Yelahanka, Karnataka, P.O. Box 6450, Bengaluru 560119, India; sudeepcellculture@gmail.com (S.N.); shraddhabijalwan1998@gmail.com (S.B.); chethuhb@gmail.com (C.K.H.B.); sbshivachandra@gmail.com (S.B.S.); brgulati@gmail.com (B.R.G.)

**Keywords:** ELISA, LSDV, risk factors, seroprevalence, whole virus antigen

## Abstract

Lumpy Skin Disease (LSD) is a transboundary bovine viral disease. It has a significant economic impact and is caused by the Lumpy Skin Disease Virus (LSDV). Effective surveillance tools are essential for the early detection of infection, outbreak control, and assessment of vaccination coverage in endemic regions such as India. In this study, an in-house ELISA based on inactivated whole-virus antigen (WVA) was developed, optimized, and validated for the detection of LSDV antibodies in cattle. Its field applicability was assessed through a cross-sectional seroprevalence survey conducted across five Indian states. A local field isolate of LSDV (strain 5-Chitra) was cultured in MDBK cells, inactivated using binary ethylenimine (BEI), and used as the antigen source. The assay was optimized by checkerboard titration and evaluated against the Serum Neutralization Test (SNT). Diagnostic sensitivity and specificity were evaluated using the receiver operating characteristic (ROC) curve and area under the curve (AUC) analyses, while cross-reactivity was assessed using sera positive for HS, IBR, BQ, MCF, GTP, SPP, CE, FMD, and Brucellosis. Assay reproducibility was confirmed through inter- and intra-laboratory validation. For the seroprevalence study, 3230 cattle serum samples were collected using a stratified random sampling design across five Indian states, and logistic regression analysis of a subset of 1302 samples was performed to assess the influence of age and sex on LSDV seropositivity. Checkerboard titration identified optimal ELISA conditions at 50 ng of antigen per well, a 1:150 serum dilution, and a 1:10,000 dilution of anti-bovine HRP-conjugated secondary antibody. The WVA-ELISA demonstrated excellent diagnostic performance, with 100% sensitivity, 95% specificity, and no cross-reactivity with other ruminant bacterial or viral pathogens, and showed high laboratory reproducibility (κ > 0.96). Seroprevalence ranged from 50.6% to 71.1% across the five states, indicating widespread exposure to LSDV. Risk factor analysis revealed significantly higher odds of seropositivity among calves (≤1 year old) and female cattle, suggesting age- and sex-dependent susceptibility.

## 1. Introduction

Lumpy skin disease (LSD) is a highly contagious viral disease of cattle caused by the lumpy skin disease virus (LSDV), a member of the genus *Capripoxvirus* within the family *Poxviridae.* The disease has major economic consequences due to its severe impact on animal health, productivity, and international trade. Outbreaks are commonly associated with reduced milk yield, weight loss, infertility, and permanent damage to hides, resulting in substantial financial losses to the livestock sector. LSDV is a double-stranded DNA virus closely related to sheep pox and goat pox viruses, sharing more than 90% genomic sequence homology, reflecting their close evolutionary relationship [1,2,3]. Clinically, LSD is characterized by fever, generalized skin nodules, and mucosal lesions, and transmission occurs predominantly through blood-feeding arthropod vectors, enabling rapid spread within and between herds [4].

Since its first description in Zambia in 1929, LSD has expanded from Africa to the Middle East, Asia, and Europe, posing a persistent global threat to cattle production systems. In India, national epidemiological data indicate LSD incidence rates ranging from 11.9% to 44.8% in indigenous cattle and from 14.3% to 45.9% in crossbred populations, with mortality rates of 0.9–16.3% and 2.5–8.4%, respectively. The impact has been particularly severe in dairy cattle, resulting in a sharp decline in milk production, currently estimated at 221.1 million tons, and projected economic losses of approximately USD 2.44 billion during 2022–2023 [5,6,7].

Accurate and timely diagnosis is essential for the effective prevention and control of LSD. Molecular diagnostic techniques, including conventional PCR and real-time PCR, provide high sensitivity and specificity; however, their reliance on specialized laboratory infrastructure, trained personnel, and relatively high operational costs limits their applicability for large-scale surveillance and field-level monitoring, particularly in resource-constrained settings [1,8].

In contrast, serological assays—most notably enzyme-linked immunosorbent assays (ELISAs)—have become indispensable tools for detecting LSDV-specific antibodies, evaluating vaccination coverage, and conducting epidemiological surveillance. ELISA-based assays offer advantages in terms of high-throughput capacity, cost-effectiveness, and operational simplicity, enabling reliable detection of anti-LSDV antibodies in both naturally infected and vaccinated animals [9,10,11]. Recent methodological advances, including the incorporation of monoclonal antibodies, recombinant antigens, and DIVA (differentiating infected from vaccinated animals)-compatible assay formats, have further improved diagnostic accuracy and strengthened their utility in disease control programs [12,13,14].

In addition to serum-based testing, milk has been explored as an alternative, non-invasive diagnostic matrix. Individual cow milk samples tested using commercial ELISAs have demonstrated high specificity and good sensitivity for the detection of LSDV antibodies, particularly when assay incubation conditions are optimized. Although bulk milk testing is comparatively less sensitive, it provides a practical and cost-effective approach for herd-level monitoring of humoral responses following LSD vaccination. Nevertheless, serum remains the most reliable matrix for accurate antibody detection at the individual animal level [15].

Seroprevalence studies play a critical role in understanding the extent of LSDV circulation within animal populations and in identifying key epidemiological risk factors. Several demographic and management-related factors, including age, breed, herd size, grazing systems, and the introduction of new animals into herds, have been reported to influence LSD seropositivity [16].

In this context, the present study aimed to develop and validate a WVA-ELISA for the detection of LSDV antibodies in cattle. The diagnostic performance, specificity, reproducibility, and cross-reactivity of the assay were evaluated in comparison with the serum neutralization test. In addition, a large-scale cross-sectional seroprevalence survey was conducted across five Indian states to estimate LSDV exposure, and logistic regression analysis was employed to identify key demographic risk factors associated with seropositivity, thereby providing valuable tools and epidemiological insights to support LSD prevalence and control strategies in endemic regions.

## 2. Materials and Methods

### 2.1. Study Design

The study was conducted in two phases. The first phase focused on the development and validation of an ELISA based on WVA for the detection of antibodies against LSDV. A reference serum panel comprising 200 SNT-positive and 200 SNT-negative samples was characterized using the serum neutralization test (SNT), which served as the reference (gold standard) assay. Analytical specificity was evaluated using sera positive for a range of viral and bacterial infections. Assay reproducibility was assessed through intra- and inter-laboratory validation studies.

The second phase consisted of a cross-sectional seroprevalence survey conducted in unvaccinated cattle across five Indian states: Karnataka, Maharashtra, Madhya Pradesh, Chhattisgarh, and Sikkim (*n* = 646 for each state). This phase also included an assessment of demographic risk factors, including age and breed (*n* = 1302), to determine their association with LSDV seropositivity.

### 2.2. Ethics Statement and Collection of Sera

Serum samples were collected from unvaccinated cattle by a local veterinarian using BD Vacutainer tubes, following approval from the respective State Departments of Animal Husbandry and Veterinary Services. Samples were transported to the laboratory in insulated containers with dry ice packs to ensure cold chain integrity. All laboratory analyses were conducted at the Indian Council of Agricultural Research -National Institute of Veterinary Epidemiology and Disease Informatics (ICAR-NIVEDI), Bengaluru, India. The study was approved by the Institute Animal Ethics Committee (Approval No. NIVEDI/IAEC/2024/06; approved on 19 March 2024).

### 2.3. Development of WVA-ELISA

#### 2.3.1. Propagation of LSDV

An in-house field isolate of LSDV, designated strain 5-Chitra and showing 100% whole-genome coverage (GenBank accession no. OR863389), was selected for this study. The virus was propagated in Madin–Darby bovine kidney (MDBK) cells (NCCS no. 2358; passage 117) obtained from the National Center for Cell Science, Pune, India. Cells were maintained under standard culture conditions, and serial passaging was performed to ensure optimal viral replication and genetic stability. All virus propagation and cell culture procedures were carried out at ICAR-NIVEDI, Bengaluru.

#### 2.3.2. Inactivation of Virus Antigen

The 12th passage of the LSDV field strain (5-Chitra) was selected for further use as the antigen. Before inactivation, it had a titre of 10^5.8^ TCID_50_/mL at a multiplicity of infection (MOI) of 0.02. The virus was inactivated using Binary Ethylenimine (BEI) following a modified protocol. Initially, the viral culture was clarified by centrifugation at 4500× *g* for 1 h at 20 °C, and the resulting supernatant was collected. A 0.3 M BEI stock solution was prepared, and its pH was adjusted to 7.6 using NaOH. BEI was then added to the viral suspension at a final concentration of 3 mM. The virus–BEI mixture was incubated at 37 °C with continuous stirring for 24 h to ensure complete inactivation. After incubation, the mixture was centrifuged at 10,000× *g* for 15 min to remove any precipitates. The supernatant was collected, and sterile 20% sodium thiosulphate solution (100 mL/L) was added to neutralize residual BEI [17]. To confirm complete inactivation, aliquots of the treated virus were subjected to three blind passages in MDBK cell lines. The final inactivated viral suspension was chilled on ice and stored at −80 °C until further use.

#### 2.3.3. Serum Neutralization Test and Preparation of Reference Serum Panels

The SNT was performed according to the standard protocol using 96-well tissue culture plates. Test sera were collected from unvaccinated cattle across five Indian states, including samples from cattle with a confirmed history of LSDV infection as well as from clinically healthy, unexposed cattle. Test sera, along with known positive and negative controls. Briefly, all sera were heat-inactivated at 56 °C for 30 min and then subjected to two-fold serial dilutions in minimum essential media (MEM). An equal volume of 100 TCID_50_ of the reference LSDV (LSDV/Cattle/India/Chitradurga/P34) was added to each serum dilution. The virus–serum mixtures were incubated at 37 °C for 1 h, after which MDBK cells suspended in growth medium (MEM supplemented with 10% Fetal bovine serum) were added to each well. Plates were incubated at 37 °C and examined daily for cytopathic effects (CPE), with final readings recorded at 96 h post-infection. For the development and optimization of the WVA-ELISA, a reference panel comprising 400 bovine serum samples with known serological status, as determined by SNT, was used.

#### 2.3.4. Optimization and Evaluation

Checkerboard titration was performed to optimize the working concentrations of the coating antigen, test sera, and conjugate according to standard ELISA protocols. Serial two-fold dilutions of the antigen and reference serum samples (SNT-positive and SNT-negative were evaluated to determine the optimal combination that yielded the highest positive-to-negative (P/N) absorbance ratio at 492 nm.

96-well microtiter ELISA plates were coated with 6–400 ng per well of inactivated LSDV WVA prepared in carbonate-bicarbonate buffer, pH 9.6 (Sigma-Aldrich, St. Louis, Missouri, USA; Cat. no. C3041), and incubated overnight at 4 °C. The plates were then washed three times with phosphate-buffered saline containing 0.05% Tween-20 (PBST), and subsequently blocked with 1% gelatin (HiMedia Laboratories, Thane, India; Cat. No. GRM019) prepared in PBST and incubated at 37 °C for 1 h. After washing, one strong positive and one negative control from the SNT reference panel were serially diluted (1:50, 1:100, 1:150, 1:200, 1:250, and 1:300) in blocking buffer. Each dilution was added in duplicate at 100 µL per well, and incubated at 37 °C for 1 h.

Following washing, 100 µL of Rabbit anti-bovine IgG horseradish peroxidase (HRPO)-conjugated antibody (Bethyl Laboratories, Montgomery, TX, USA; Cat. No. A101-02P), diluted at 1:2500; 1:5000; 1:7500; 1:10,000; 1:12,500 and 1:15,000 was added to each well and incubated at 37 °C for 1 h. After thorough washing, 100 µL of o-phenylenediamine dihydrochloride (OPD) substrate solution was added to each well. The substrate was prepared by dissolving 5 mg OPD tablets (Sigma-Aldrich; Cat. No. P6912) in 12.5 mL of distilled water containing 100 µL of 3% hydrogen peroxide. The plates were incubated at 37 °C for 7 to 10 min in the dark without shaking, and the reaction was stopped by adding 50 µL of 1 M sulfuric acid. Optical density (OD) was measured at 492 nm using a microplate reader (Tecan Infinite F50, Männedorf, Switzerland). For each sample, the percentage positivity value (PPV) was calculated using the formula:

$$PPV=\frac{{OD}_{TS}-{OD}_{NC}}{{OD}_{PC}-{OD}_{NC}}\times 100$$

where OD_TS_ represents the optical density of the test sample, OD_PC_ represents the optical density of the positive control, and OD_NC_ corresponds to the optical density of the negative control. Samples with a PPV of 35% or less were classified as negative, whereas those with a PPV greater than 35% were considered positive.

#### 2.3.5. Cross Reactivity, Sensitivity, Specificity, and Validation

The cross-reactivity of the developed WVA-ELISA was assessed using heterologous serum samples previously confirmed positive for Contagious Ecthyma (CE), Foot-and-Mouth Disease (FMD), Hemorrhagic Septicemia (HS), Brucellosis, Infectious Bovine Rhinotracheitis (IBR), Malignant Catarrhal Fever (MCF), Black Quarter (BQ), Goat Pox (GTP), and Sheep Pox (SPP). All the sera were additionally tested by the SNT assay and confirmed to be negative for LSDV-specific antibodies. These analyses were performed to confirm the absence of cross-reactivity and to ensure antigenic specificity of the assay for LSDV.

To determine the diagnostic sensitivity and specificity of the WVA-ELISA, a panel of 400 bovine serum samples previously characterized as positive or negative by SNT was analyzed. The ELISA results were compared with those of the SNT, which was used as the reference (gold standard) test. Receiver Operating Characteristic (ROC) curve analysis was performed using MedCalc software (version 12.5.0.0; MedCalc Software, Mariakerke, Belgium). The area under the curve (AUC), positive and negative likelihood ratios, and the optimal ELISA cut-off value were calculated from this analysis [18].

Assay repeatability and precision were assessed by calculating the coefficient of variation (CV), defined as the ratio of the standard deviation to the mean optical density (OD) of replicate measurements.

Furthermore, the WVA-ELISA was validated for inter- and intra-laboratory reproducibility through independent testing by three analysts at different institutes to evaluate the robustness, stability, and reproducibility of the assay.

### 2.4. Seroprevalence Study with Associated Risk Factors

State-wise seroprevalence of LSD was estimated based on seropositivity data obtained using the developed WVA-ELISA in five Indian states: Karnataka, Maharashtra, Chhattisgarh, Madhya Pradesh, and Sikkim. Spatial visualization of seroprevalence was performed using maps generated with Quantum Geographic Information System (QGIS) software (version 3.32.2).

The required sample size for estimating LSD seroprevalence in cattle from every five Indian states was calculated using the standard formula available in Epitools (https://epitools.ausvet.com.au/, accessed on 5 october 2025), an online epidemiological calculator. Specifically, the sample size was determined using the method “sample size to estimate a proportion or apparent prevalence with specified precision,” which is designed to estimate a population proportion with a predefined level of precision. The following standard formula was applied:

$$n=\frac{\left[Z^{2}\times p\times \left(1-p\right)\right]}{d^{2}}$$

where

n = required sample size

Z = Z-value for 95% confidence level (1.96)

p = expected seroprevalence (assumed at 0.3, based on prior LSD studies)

d = desired precision (0.05)

Substituting the values:

$$n=\frac{{\left(1.96\right)}^{2}\times 0.3\times (1-0.3)}{{\left(0.05\right)}^{2}}=\frac{3.8416\times 0.21}{0.0025}=\frac{0.8067}{0.0025}=322.68$$

Accordingly, a minimum of 323 samples per state was deemed sufficient to estimate seroprevalence with 95% confidence and ±5% precision. To account for potential intra-cluster correlation arising from sampling across multiple villages or herds, a design effect (DEFF) of 2 was applied for cluster sampling. This adjustment increased the target sample size to 646 samples per state, resulting in a total of 3230 samples across all five states. A stratified random sampling approach was used to ensure representative coverage within each state.

Statistical analyses were performed using IBM Statistics software version 27. Complete demographic data were obtained for 1302 of the 3230 serum samples collected, all of which were obtained from unvaccinated exotic crossbred cattle. Seropositivity determined by the developed WVA-ELISA was considered the dependent variable. Age and sex were included as independent variables based on their presumed association with LSDV seropositivity. Age was categorized into four groups: ≤1 year (n = 326), 2–4 years (n = 326), 5–7 years (n = 326), and >7 years (n = 324). Sex was divided as female (n = 651) and male (n = 651). Logistic regression models were fitted to estimate odds ratios (ORs), expressed as Exp(B), to quantify the strength and direction of associations between predictors and seropositivity. The precision of the estimates was evaluated using 95% confidence intervals (CIs). Statistical significance was assessed using two-tailed Wald test *p*-values, with *p* < 0.05 considered statistically significant. Variables with *p* ≥ 0.05 were regarded as not statistically significant [19].

## 3. Results

### 3.1. Development and Standardization of WVA-ELISA

#### 3.1.1. Propagation and Inactivation of LSDV

**Figure 1:**
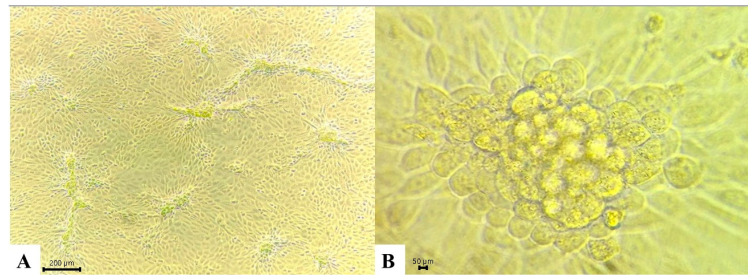
CPE in MDBK cells infected with LSDV. MDBK cell line infected with 5-Chitra strain of LSDV after 96 hrs. (**A**) Infected cells exhibiting rounding and clumping on the surface as CPE at 10×; (**B**) Magnified view at 40× showing distinct cellular rounding and aggregation, with loss of cellular architecture and integrity, consistent with progressive viral replication and damage.

The LSDV field isolate 5-Chitra was propagated in MDBK cells under bulk culture conditions. The cultures were monitored daily for the development of CPE. The plaque-like CPE typical of cells 96 h post-LSDV-infection is shown in Figure 1.

The harvested virus was inactivated by using BEI and clarified by centrifugation. This final WVA preparation did not show any CPE after 3 blind passages in MDBK cells. The inactivated virus did not show any CPE in all the 3 blind passages.

#### 3.1.2. Serum Neutralization Test and Preparation of Reference Serum Panels

Samples with SNT titers ≥ 1:8 were classified as LSDV antibody-positive (n = 200), while those with titers < 1:8 were classified as antibody-negative (n = 200) and were taken as reference panels. SNT-negative and SNT-positive titers are depicted in Table 1. These sera were used for assay optimization, including the determination of diagnostic cut-off values and performance evaluation of the WVA-ELISA.

#### 3.1.3. Optimization and Evaluation

**Figure 2:**
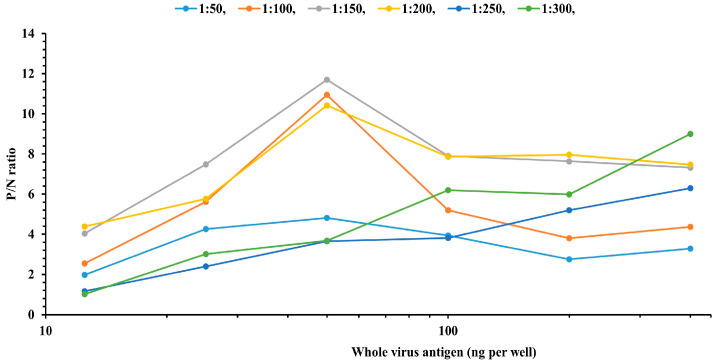
Optimization of LSDV ELISA conditions using checkerboard titration. Plots depict optimization at a 1:10,000 dilution of HRP-conjugated secondary antibody using two-fold serial dilutions of WVA tested against serial serum dilutions. Peak P/N ratios at intermediate antigen concentrations indicate optimal coating conditions.

Optimization by checkerboard titration demonstrated that an antigen concentration of 50 ng/well, a serum dilution of 1:150 (Figure 2), and a rabbit anti-bovine HRP-conjugated secondary antibody dilution of 1:10,000 provided the optimal conditions for the detection of LSDV-specific antibodies. The detailed checkerboard titration results for the anti-bovine HRP-conjugated secondary antibody are presented in Supplementary Table S1. Using these optimized assay conditions, a panel of 400 reference serum samples with known positive and negative status was screened, and the results were expressed as PPV.

#### 3.1.4. Cross Reactivity, Sensitivity, and Specificity

The developed WVA-ELISA showed no cross-reactivity with sera positive for other viral infections, such as CE, FMD, SPP, GTP, IBR, and MCF, or with bacterial diseases, such as Brucellosis, BQ, and HS. All heterologous sera yielded PPV below the established 35% cut-off (Supplementary Table S2).

**Figure 3:**
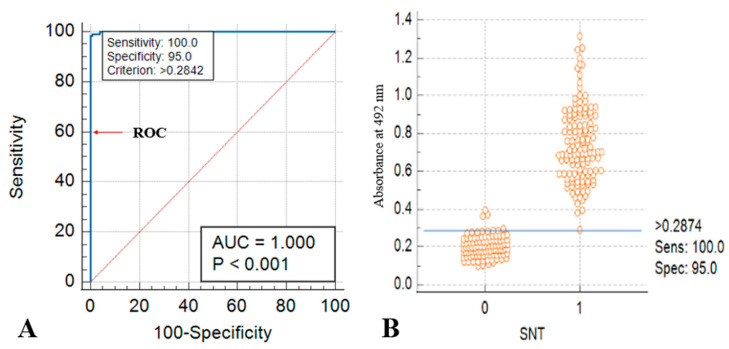
Evaluation of diagnostic specificity and sensitivity of WVA-ELISA for LSDV detection in cattle. (**A**) ROC curve illustrating the diagnostic performance of the developed whole virus antigen ELISA for detecting LSDV-specific antibodies in sera. (**B**) Scatter plot comparing ELISA values against results from SNT (SNT-positive (1) and SNT-negative (0)).

Diagnostic specificity and sensitivity were further evaluated using ROC and AUC analyses with known positive and negative sera. The analysis yielded an AUC of 1.000 (*p* < 0.001), with a sensitivity of 100% and specificity of 95% at the optimal cut-off value (Figure 3A).

A scatter plot comparing ELISA optical density (OD) values with SNT results demonstrated a clear separation between positive and negative samples. Sera positive by SNT (value = 1) showed higher ELISA OD values, whereas the majority of SNT-negative sera showed OD values below the established ELISA cut-off threshold (0.2874). However, a limited number of samples had marginally elevated OD values, which likely reflect low-level background reactivity rather than true seropositivity. (Figure 3B). Inter- and intra-institutional validation of the assay demonstrated reproducibility, with mean Cohen’s kappa values of 0.966 and 0.962, respectively.

### 3.2. Seroprevalence Study with Associated Risk Factors

**Figure 4:**
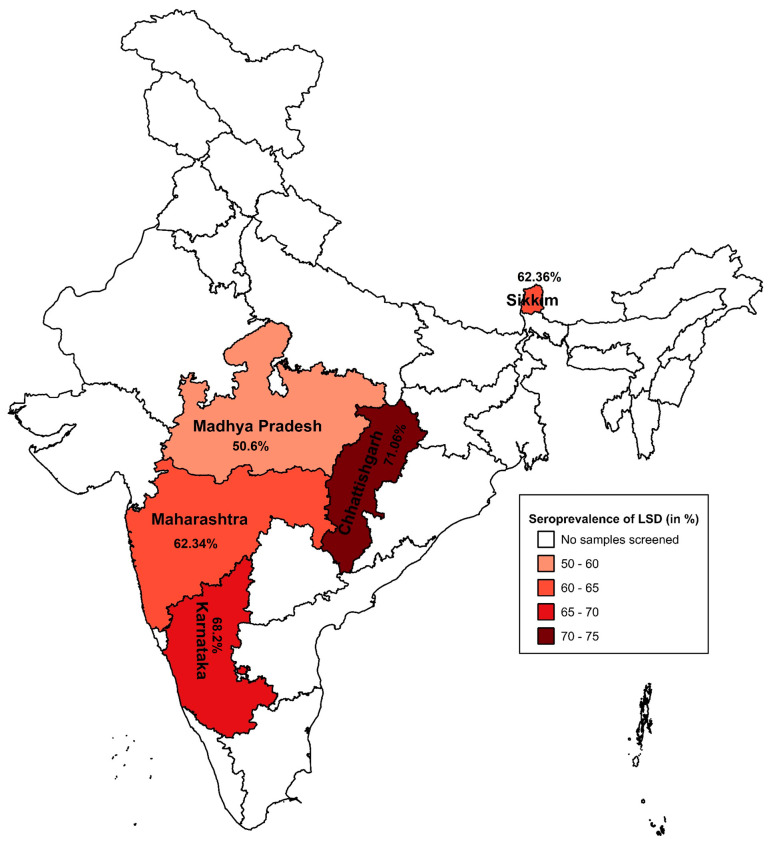
Seroprevalence of LSD in India. Seroprevalence of LSD is presented as percentage ranges, color-coded from light to dark red, for the five states with available seroprevalence data.

The seroprevalence of LSD in five surveyed states, with Chhattisgarh reporting the highest prevalence at 71.06%, followed by Karnataka (68.2%), Sikkim (62.36%), Maharashtra (62.34%), and Madhya Pradesh (50.6%) (Figure 4).

Univariate logistic regression analysis revealed that both age and sex were significantly associated with seropositivity. Cattle aged ≤ 1 year had 2.3-fold higher odds of being seropositive (OR = 2.399, 95% CI: 1.747–3.293) compared to the reference group (>7 years). Cattle aged 2–4 years and 5–7 years showed 74.7% (OR = 1.747, 95% CI: 1.254–2.361) and 25.4% (OR = 1.254, 95% CI: 0.975–1.844) increased odds of seropositivity, respectively. Female cattle showed 28.3% higher odds of seropositivity compared to males (OR = 1.283, 95% CI: 1.031–1.598) (Table 2).

Multivariate logistic regression confirmed that both age and sex were significant predictors of LSD occurrence in cattle (overall *p* < 0.05). Female cattle had significantly higher odds of infection than males (OR = 1.57, 95% CI: 1.243–1.982). Age remained a strong predictor, with cattle aged ≤ 1 year showing the highest odds of infection (OR = 2.862, 95% CI: 2.051–3.992), followed by those aged 2–4 years (OR = 1.936, 95% CI: 1.400–2.679) and 5–7 years (OR = 1.517, 95% CI: 1.094–2.104), relative to the reference group of cattle older than 7 years (Supplementary Table S3).

## 4. Discussion

The SNT is widely regarded as the reference method for detecting LSDV-specific antibodies due to its ability to measure functional virus-neutralizing activity. However, its application in large-scale sero-surveillance and epidemiological studies is limited by several practical constraints. The SNT requires the handling of live viruses and continuous cell culture systems, necessitating high-level biosafety infrastructure, specialized technical expertise, and extended assay durations, often ranging from several days to more than one week. These factors significantly restrict the feasibility for high-throughput testing, particularly in resource-limited and LSD-endemic settings [9,20].

In contrast, ELISAs offer a safer, faster, and more scalable alternative for antibody detection. The choice of coating antigen is a critical determinant of ELISA performance, directly influencing assay sensitivity and specificity. Since the emergence of LSD in India in 2019 and its substantial economic impact on the livestock sector, there has been an increasing demand for reliable serological tools to support prevalence studies, surveillance, and control programs. Although commercial ELISA kits are available, their high cost and reliance on imported reagents often limit accessibility, particularly for large-scale and routine monitoring in low-resource settings, highlighting the need for affordable, locally produced, and diagnostically robust in-house assays [6,21].

In this study, we developed and optimized an in-house ELISA based on inactivated WVA derived from a locally circulating LSDV strain. The optimized assay conditions: 50 ng of antigen per well, 1:150 serum dilution, and 1:10,000 dilution of anti-bovine HRP-conjugated secondary antibody resulted in excellent diagnostic performance, with 100% sensitivity and 95% specificity relative to the SNT. These findings are consistent with previous reports, such as Sthitmatee et al. [9], who developed an in-house ELISA achieving high sensitivity (94.9%) and specificity (89.8%). Notably, the sensitivity of the present assay exceeds that reported for some commercial kits (91.3%), while specificity remains comparable (commercial ELISA: 91.4%). Collectively, these results demonstrate that appropriately optimized in-house ELISAs can provide diagnostic performance comparable to—or, in some aspects, exceeding—that of commercial assays, highlighting their suitability for serosurveillance and large-scale epidemiological studies.

The state-wise differences in seroprevalence observed in this study reflect regional heterogeneity in LSDV transmission dynamics. The validated WVA-ELISA was successfully applied in a cross-sectional seroprevalence survey of unvaccinated exotic cattle across five Indian states, revealing high seroprevalence in Chhattisgarh, Karnataka, Sikkim, and Maharashtra, while Madhya Pradesh exhibited comparatively lower seropositivity. These findings suggest that recent outbreak history, climatic conditions favorable for vector proliferation, and regional management practices may contribute to differences in virus exposure and transmission dynamics. Similar patterns have been reported in other LSDV-endemic regions; for instance, Ochwo et al. [16] documented widespread herd-level infection in Uganda, with higher seropositivity associated with areas of increased rainfall, humidity, and intensively managed farms practicing communal watering. These environmental and management-related factors likely enhance animal contact rates and promote the proliferation of mechanical vectors, facilitating sustained virus transmission. The detection of substantial seropositivity in regions with relatively low reported clinical morbidity underscores the importance of serological surveillance for identifying subclinical infections and silent virus circulation that are not captured through clinical reporting alone [22,23]. Interestingly, even in states with lower clinical morbidity, such as Madhya Pradesh, notable levels of seropositivity were observed, suggesting the occurrence of subclinical infections or previous virus exposure that may go undetected through conventional clinical surveillance. These results highlight the limitations of relying solely on reported clinical cases for assessing LSDV circulation and emphasize the critical role of sero-epidemiological studies in informing disease control and vaccination strategies [24].

Consistent with previous reports, age and sex were identified as significant predictors of LSDV seropositivity. The significantly higher seropositivity observed in cattle ≤1 year of age indicates increased susceptibility in younger animals. Younger cattle (≤1 year) exhibited the highest risk of infection, likely due to an immature immune system, and increased susceptibility to insect vectors, which preferentially feed on calves and young animals due to thinner skin and reduced grooming behavior [25]. The higher odds of seropositivity in females observed in this study are consistent with prolonged herd retention and cumulative exposure. Additionally, physiological factors, including hormonal fluctuations and stress associated with pregnancy and lactation, may further modulate immune responses, increasing susceptibility [16,19,26,27]. These findings have important epidemiological and management implications, suggesting that vaccination and vector control strategies should be prioritized for young and female animals, particularly during periods of high vector activity. Awareness programs emphasizing effective vector management, restriction of animal movement during outbreaks, and timely vaccination could further reduce virus transmission in endemic regions.

Recent developments in ELISA design highlight the evolving landscape of LSDV serodiagnosis. Differentiating Infected from Vaccinated Animals (DIVA) ELISAs provide the ability to distinguish between vaccine-induced and infection-induced antibodies, addressing a key limitation of conventional ELISA formats in post-vaccination surveillance [14]. Although the current WVA-ELISA does not incorporate DIVA capability, such approaches represent a promising direction for future assay refinement, particularly in regions implementing widespread vaccination campaigns. Similarly, competitive ELISAs targeting conserved viral epitopes enable antibody detection across multiple host species, increasing the utility of serological testing for multi-species surveillance in mixed livestock systems [12]. Future research could focus on adapting the WVA-ELISA for additional susceptible species, such as yak and Mithun, through the use of species-specific secondary antibodies or conjugates, thereby broadening its diagnostic applicability and supporting cross-species monitoring of LSDV.

Due to resource constraints, direct comparison of the WVA-ELISA with commercial kits could not be performed, limiting the evaluation of relative performance against proprietary reagents. Additionally, the available demographic information was restricted to vaccination status, age, breed, and sex, while data on herd management practices, animal movement, vector control measures, and herd size were not collected. Consequently, the influence of these additional epidemiological and management-related factors on LSDV seropositivity could not be fully assessed. Addressing these gaps in future studies through expanded datasets, comparative assessments with commercial assays, and inclusion of broader risk factors would strengthen the epidemiological insights and enhance the application of in-house ELISA platforms for targeted disease control and vaccination strategies.

## Figures and Tables

**Table 1:** Distribution of SNT titers in the reference panel. The table summarizes the distribution of serum neutralization test (SNT) antibody titers in the reference serum panel used for assay validation. Samples with titers < 1:8 were classified as SNT-negative, whereas samples with titers ≥ 1:8 were considered SNT-positive and indicative of the presence of LSDV-neutralizing antibodies.

SNT Titer	No. of Samples	Reference Panel
1:2	29	SNT negative
1:4	171	
1:8	26	SNT positive
1:16	66	
1:32	60	
1:64	47	
1:128	1	

**Table 2:** Univariate regression analysis of LSD with demographic factors. The table shows univariate logistic regression performed for the association between gender and age with the likelihood of LSD occurrence in cattle.

Risk Factors		OR	95% CI	*p*-Value
Gender	Female	1.283	1.031–1.598	0.026 *
	Male (Reference)	1	-	-
Age	Overall *p*-value			<0.001 *
	≤1 year	2.399	1.747–3.293	<0.001 *
	2–4 years	1.721	1.254–2.361	0.001 *
	5–7 years	1.341	0.975–1.844	0.072
	>7 years(Reference)	1	-	-

Abbreviations: * *p* < 0.05 = significant, OR = odds ratio, and CI = confidence interval (Lower Limit-Upper Limit).

## Data Availability

The raw data supporting the conclusions of this article will be made available by the authors on request.

## Supplementary Materials

The following supporting information can be downloaded at: https://www.mdpi.com/article/10.3390/microorganisms14020373/s1, Table S1. Optimization of serum and conjugate dilutions for WVA-ELISA by checkerboard titration. ELISA optimization was carried out using 50 ng/well of WVA antigen. OD values obtained for cattle positive and negative sera across different serum and conjugate dilutions are shown. Table S2. Cross-reactivity of WVA-ELISA assessed using heterologous reference sera. Reference positive sera (*n* = 3 per disease) for selected bovine and small ruminant diseases obtained from ICAR-NIVEDI were tested to evaluate cross-reactivity. Table S3. Multivariate regression analysis of LSD with risk factors. The table shows multivariate logistic regression to show the association between the risk factors with the likelihood of LSD occurrence in cattle.
