# Supplementary material for: Development and Validation of a Population Assay for the Seroprevalence of Lumpy Skin Disease
Source: Microorganisms. 2026 Feb 5;14(2):373. doi: 10.3390/microorganisms14020373 (PMC12942835; doi:10.3390/microorganisms14020373)
Supplement: Supplementary file 1 [file microorganisms-14-00373-s001.zip › microorganisms-4066737-supplementary.pdf]

SUPPLEMENTARY TABLES

Table S1. Optimization of serum and conjugate dilutions for WVA-ELISA by checkerboard titration. ELISA optimization was carried out using 50 ng/well of WVA antigen. OD values obtained for cattle positive and negative sera across different serum and conjugate dilutions are shown.

Dilutions	Cattle Positive sera						Cattle Negative sera					
	Conjugate dilution						Conjugate dilution					
	1:2,500	1:5,000	1:7,500	1:10,000	1:12,500	1:15,000	1:2,500	1:5,000	1:7,500	1:10,000	1:12,500	1:15,000
**1:50**	2.802	1.8576	1.8526	1.8406	0.4752	0.3312	0.7668	0.6732	0.369	0.2746	0.2116	0.1674
**1:100**	2.712	1.6523	1.7874	1.843	0.576	0.279	0.558	0.5364	0.3438	0.2044	0.1926	0.1098
**1:150**	2.708	1.7259	1.7608	1.78	0.656	0.3564	0.333	0.3042	0.2862	0.122	0.098	0.0882
**1:200**	2.298	1.562	1.386	1.0314	0.5598	0.3312	0.2772	0.2466	0.1764	0.199	0.0972	0.0756
**1:250**	1.638	1.179	0.6246	0.342	0.2196	0.1422	0.234	0.2268	0.1638	0.0936	0.0918	0.1224
**1:300**	0.936	0.5706	0.513	0.3312	0.3204	0.126	0.0936	0.0954	0.0828	0.09	0.1062	0.1242

Table S2. Cross-reactivity of WVA-ELISA assessed using heterologous reference sera. Reference positive sera (*n* = 3 per disease) for selected bovine and small ruminant diseases obtained from ICAR-NIVEDI were tested to evaluate cross-reactivity.

Sl. No	Disease	No. of samples tested	PPV1	PPV2	PPV3	Mean PPV
1	HS*	3	32.1	34.9	34.5	33.83
2	Brucellosis	3	3.321	3.542	3.472	3.445
3	IBR*	3	3.124	2.044	2.224	2.464
3	FMD*	3	10.082	13.923	14.875	12.96
4	BQ*	3	2.981	1.695	2.716	2.464
6	MCF*	3	10.742	8.421	8.554	9.293
7	GTP*	3	14.87	15.869	16.791	15.843
8	SPP*	3	14.435	16.819	14.795	15.34
9	CE*	3	2.026	2.794	2.536	2.45

*HS, Hemorrhagic septicemia; IBR, Infectious bovine rhinotracheitis; FMD, Foot-and-mouth disease; BQ, Black quarter; MCF, Malignant catarrhal fever; GTP, Goatpox ; SPP, Sheeppox ; CE, Contagious Ecthyma.

Table S3. Multivariate regression analysis of LSD with risk factors**.** The table shows multivariate logistic regression to show the association between the risk factors with the likelihood of LSD occurrence in cattle.

**Risk factors**		**OR**	**95% CI**	**p-value**
Overall p value				< 0.001*
Gender	Female	1.57	1.243-1.982	< 0.001*
	Male	1	-	-
Age	≤1 year	2.862	2.051-3.992	< 0.001*
	2-4 years	1.936	1.400-2.679	< 0.001*
	5-7 years	1.517	1.094-2.104	0.013*
	> 7 years	1	-	-

Abbreviations: * $p < 0.05$ = significant, OR = odds ratio, and CI = confidence interval.
